# Centile charts for birthweight for gestational age for Scottish singleton births

**DOI:** 10.1186/1471-2393-8-5

**Published:** 2008-02-25

**Authors:** Sandra Bonellie, James Chalmers, Ron Gray, Ian Greer, Stephen Jarvis, Claire Williams

**Affiliations:** 1 School of Accountancy, Economics and Statistics, Napier University, 10 Colinton Road, Edinburgh, EH10 5DT, UK; 2 ISD, Gyle Square, 1 South Gyle Crescent, Edinburgh, EH12 9EB, UK; 3 NPEU, University of Oxford, Old Road Campus, Headington, Oxford, OX3 7LF, UK; 4 Hull York Medical School, University of York, York, UK; 5 Sir James Spence Institute of Child Health, Royal Victoria Infirmary, Queen Victoria Road, Newcastle Upon Tyne, NE1 4LP, UK

## Abstract

**Background:**

Centile charts of birthweight for gestational age are used to identify low birthweight babies. The charts currently used in Scotland are based on data from the 1970s and require updating given changes in birthweight and in the measurement of gestational age since then.

**Methods:**

Routinely collected data of 100,133 singleton births occurring in Scotland from 1998–2003 were used to construct new centile charts using the LMS method.

**Results:**

Centile charts for birthweight for sex and parity groupings were constructed for singleton birth and compared to existing charts used in Scottish hospitals.

**Conclusion:**

Mean birthweight has been shown to have increased over recent decades. The differences shown between the new and currently used centiles confirm the need for more up-to-date centiles for birthweight for gestational age.

## Background

Birthweight is one of the important indicators used to assess the health of an infant at birth. Low birthweight has often been defined as weights less than 2500 grams with birthweights less than 1500 grams classed as very low birthweight. These definitions however do not take into account gestational age. It is important to be able to differentiate between babies who are light because they are premature and those who are small-for-gestational age since the latter may have different health problems to the former. They may be growth restricted and have an increased risk of other complications such as perinatal asphyxia, symptomatic hypoglycaemia, congenital malformations, chronic intra-uterine infection and pulmonary haemorrhage [1]. Large-for-gestational age babies also have related health problems. Identification of small or large for gestational age babies is important for the management of the individual pregnancy and neonate. It is also a valuable aid to epidemiological studies where the aim is to identify risk factors or to assess the management of pregnancies [2].

Small- or large-for-gestational age babies may be identified using centile charts of birthweight by gestational age. Centile reference charts are used to monitor clinical measurements on individuals in the context of population values. Raw centiles can be calculated from appropriate data but the perturbations in these curves are unlikely to reflect the pattern of underlying growth at the population level. It is therefore reasonable to use statistical methods to derive a series of smoothed curves showing how the centiles of a measurement, in this case birthweight, change when plotted against time, in this case gestational age.

In Scotland there have been three sizeable studies resulting in the production of centile charts each based on data collected in Aberdeen [3-5]. The charts from the most recent of these studies, using data from 17,927 singleton births occurring between 1975–1980, were extensively used as a standard throughout Scotland until relatively recently.

The Information Services Division (ISD) of the Scottish Health Service use and publish birthweight centile charts[6]. ISD collects data on all maternity patients admitted to Scottish hospitals on an SMR02 form. The charts are based on 894,066 live births occurring between 1975 and 1989 and are the most recent published in Scotland.

Increases in birthweight since the formation of these standards have been observed for Scotland [7] England and Wales [8], the United States [9] and Canada [10] There have also been changes in the methods used to calculate gestational age [11]. These changes suggest that the centile charts in present use may now be inaccurate. Therefore, we aimed to produce updated charts using more recent data from 1998–2003.

## Methods

Data on singleton births occurring between 1980 and 2003 were obtained from ISD's SMR02 (maternity) data collection system. This includes information on the birthweight, gestational age and sex of the infant. The parity of the mother is also recorded. Gestational age at birth was reported in completed weeks and is a clinician's estimate of gestation at birth based on an ultrasound dating scan and date of last menstrual period.

In order to adequately represent the population of all singleton births, the only exclusions made were lethal congenital anomalies and obvious outliers which included any birthweights less than 250 grams. Outliers were identified using Tukey's methodology [12]. This calculates the interquartile range and identifies as outliers any values more than twice the interquartile range below the first quartile or above the third quartile. This method assumes a symmetric distribution which is not the case for birthweights at most gestational ages. However the values of L obtained in each of the groups for each suggests only a slight degree of skewness at most gestational ages. The number of birthweights omitted as possible outliers was small and inspection of the omitted birthweights suggests that most of these could be explained by transcription errors.

The mean birthweight of all singletons born in each year between 1980–2003 was calculated. This confirmed the reported increase in birthweight over this period. This increase is marked over the period from 1980–1997 but appears to level off from 1998 onwards and therefore the most recent years for which complete data were available, namely 2002 and 2003, were used as a basis from which to construct new centile charts. For births occurring at gestations between 31 and 42 weeks a two year period gives sufficient data, however for the extremes of gestational age the data was supplemented by births from 1998 to 2001.

Centiles were calculated using the LMS method [13] which uses the Box-Cox power transformation to obtain normally distributed data within each group. This involves estimating three sets of values for each gestational age group, namely, L the power transformation used to achieve normality, M the median birthweight and S the coefficient of variation of the data. L, M and S are estimated for each gestational age and then smoothed curves are fitted using cubic splines to these to give L(t), M(t) and S(t) where t is the gestational age. The extent of the smoothing is expressed in terms of the degrees of freedom used for the fit. The 100*α*th centile for the appropriate sex and parity group is then given by

C_100*α *_(t) = M(t) [1+L(t)S(t)Z_*α*_]^1/*L*(*t*)^

where Z_*α *_is the *α *% point of the normal distribution.

For a particular infant, with birthweight y, a z-score can be calculated using the formula

$$\text{z}=\frac{{\left[\frac{\text{y}}{\text{M}\left(\text{t}\right)}\right]}^{\text{L}\left(\text{t}\right)}-1}{\text{L}\left(\text{t}\right)\text{S}\left(\text{t}\right)}$$

Four sets of charts were constructed defined by the sex of the baby, male or female, and the parity of the mother, nulliparous or multiparous. Centiles were calculated using the software LMS ChartMaker. Other analysis was carried out using SAS, version 9.1

## Results

**Figure 1:**
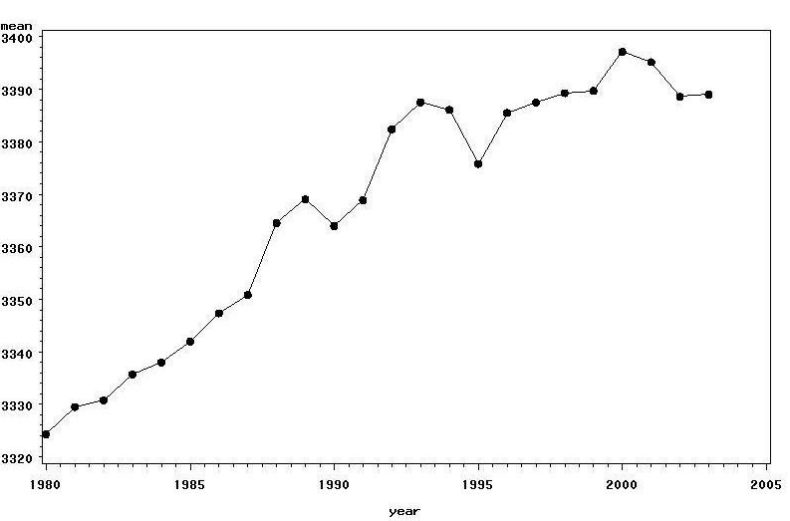
Mean birthweight by year.

The mean birthweight for each of the years from 1980 to 2003 is shown in Figure 1 and confirms the previously reported increase in birthweight.

**Figure 2:**
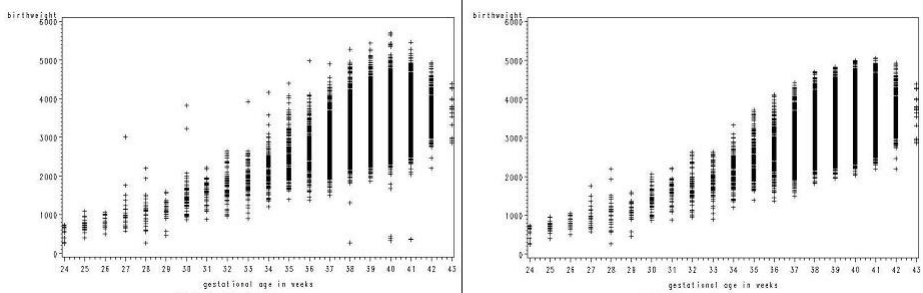
**a: Birthweight by gestational age for girls, parity 1 or more.** b: Birthweight by gestational age for girls, parity 1 or more with outliers removed.

There were 98,904 records of singleton births occurring in 2002 and 2003. These were supplemented by information on 1,883 singleton births from 1998–2001 for gestational ages of 30 weeks or less or 43 weeks. Excluding lethal congenital anomalies and omitting outliers gave a total of 100,133 records. Applying Tukey's method resulted in 0.4% of the observations being omitted as outliers. Figures 2a and 2b show plots of birthweight against gestational age with and without the outliers for the subgroup girls, parity 1 or more. Table 1 gives the numbers of births used in constructing the centiles, and the percentage of outliers omitted together with the overall mean birthweight and standard deviation based on the data for 2002–2003 only.

**Table 1:** Summary of Data by Sex and Parity Groupings

**Group**		**Total Numbers**		**2002–2003 Data**
**Sex of infant**	**Parity**	**Number of births used**	**Percentage of outliers**	**Mean(St.Dev) Birthweight (with outliers omitted)**

**Male**	**0**	23419	0.37	3376 (603.33)
	**1 or more**	27924	0.43	3494 (603.03)
**Female**	**0**	21948	0.39	3266 (570.94)
	**1 or more**	26842	0.37	3369 (570.79)

Tables 2, 3, 4, 5 give the centiles for the groups: boys parity 0, boys parity 1 or more, girls parity 0 and girls parity 1 or more respectively. The tables also give the number of births used and the fitted values of L. M and S for each gestational age for each group, as well as the degrees of freedom used in fitting the cubic splines.

**Table 2:** Centiles for Boys, Nulliparous

**Gestational Age**	**No**.	**L d.f. = 5**	**M d.f. = 12**	**S d.f. = 6**	**3^rd^**	**5^th^**	**10^th^**	**25^th^**	**50^th^**	**75^th^**	**90^th^**	**95^th^**	**97^th^**
**24**	65	1.30	658	0.245	326	372	440	546	658	764	856	910	944
**25**	59	1.34	759	0.240	379	432	510	632	759	879	982	1042	1080
**26**	101	1.38	851	0.235	430	490	577	712	851	982	1095	1160	1202
**27**	105	1.41	958	0.229	494	561	656	805	958	1101	1224	1295	1341
**28**	128	1.43	1103	0.222	585	659	766	932	1103	1263	1400	1479	1530
**29**	143	1.44	1271	0.214	696	778	896	1081	1271	1449	1601	1689	1745
**30**	160	1.43	1446	0.205	823	911	1039	1239	1446	1640	1807	1904	1966
**31**	86	1.41	1643	0.196	976	1068	1204	1419	1643	1855	2037	2143	2211
**32**	105	1.38	1848	0.187	1142	1239	1382	1609	1848	2075	2272	2387	2460
**33**	121	1.34	2065	0.178	1326	1425	1574	1812	2065	2308	2519	2643	2722
**34**	213	1.27	2286	0.169	1521	1622	1774	2021	2286	2543	2768	2901	2986
**35**	341	1.17	2510	0.161	1729	1830	1983	2235	2510	2779	3018	3159	3250
**36**	586	1.05	2744	0.153	1950	2050	2204	2461	2744	3026	3278	3428	3526
**37**	1051	0.93	2959	0.145	2159	2259	2412	2671	2959	3250	3513	3671	3774
**38**	2447	0.84	3162	0.137	2363	2461	2613	2871	3162	3457	3726	3889	3996
**39**	4459	0.77	3341	0.130	2546	2643	2794	3050	3341	3638	3910	4075	4182
**40**	6421	0.74	3510	0.125	2711	2809	2960	3217	3510	3809	4083	4250	4359
**41**	5906	0.76	3664	0.120	2859	2957	3110	3369	3664	3964	4238	4405	4514
**42**	867	0.83	3736	0.116	2935	3034	3187	3445	3736	4031	4299	4461	4567
**43**	55	0.92	3764	0.112	2976	3074	3225	3479	3764	4050	4309	4465	4566

**Table 3:** Centiles for Boys, Multiparous

**Gestational Age**	**No**.	**L d.f. = 4**	**M d.f. = 15**	**S d.f. = 8**	**3^rd^**	**5^th^**	**10^th^**	**25^th^**	**50^th^**	**75^th^**	**90^th^**	**95^th^**	**97^th^**
**24**	61	1.24	628	0.229	339	378	436	529	628	723	806	855	886
**25**	59	1.23	756	0.224	418	463	531	640	756	868	966	1024	1061
**26**	71	1.22	866	0.218	490	540	615	736	866	991	1101	1166	1207
**27**	72	1.21	996	0.212	578	633	716	851	996	1137	1260	1333	1380
**28**	111	1.20	1147	0.207	679	740	833	984	1147	1305	1444	1526	1579
**29**	122	1.18	1308	0.203	788	856	959	1126	1308	1484	1640	1732	1791
**30**	153	1.15	1483	0.200	907	982	1096	1281	1483	1681	1856	1959	2026
**31**	62	1.10	1676	0.196	1044	1125	1249	1453	1676	1897	2093	2209	2284
**32**	100	1.05	1859	0.192	1183	1269	1400	1618	1859	2099	2314	2442	2525
**33**	135	0.98	2065	0.187	1344	1434	1573	1806	2065	2325	2560	2701	2792
**34**	209	0.90	2284	0.181	1520	1614	1760	2007	2284	2565	2821	2975	3076
**35**	312	0.82	2523	0.178	1708	1807	1961	2224	2523	2828	3108	3278	3389
**36**	679	0.75	2792	0.172	1927	2031	2194	2473	2792	3121	3425	3610	3731
**37**	1448	0.70	3063	0.160	2181	2287	2452	2737	3063	3400	3711	3902	4027
**38**	3940	0.69	3313	0.144	2457	2560	2721	2997	3313	3639	3940	4124	4245
**39**	6247	0.70	3480	0.130	2663	2762	2916	3179	3480	3788	4072	4245	4358
**40**	7809	0.73	3649	0.123	2831	2931	3086	3349	3649	3955	4236	4407	4519
**41**	5665	0.76	3793	0.120	2962	3063	3221	3489	3793	4102	4386	4557	4670
**42**	629	0.79	3856	0.120	3005	3110	3272	3546	3856	4172	4460	4634	4748
**43**	40	0.83	3866	0.123	2987	3095	3263	3547	3866	4190	4486	4665	4781

**Table 4:** Centiles for Girls, Nulliparous

**Gestational Age**	**No**.	**L d.f. = 4**	**M d.f. = 12**	**S d.f. = 6**	**3^rd^**	**5^th^**	**10^th^**	**25^th^**	**50^th^**	**75^th^**	**90^th^**	**95^th^**	**97^th^**
**24**	55	1.43	604	0.254	270	319	389	496	604	704	789	838	869
**25**	56	1.35	682	0.252	320	372	446	562	682	794	891	947	983
**26**	78	1.27	779	0.249	382	437	517	645	779	907	1018	1084	1125
**27**	69	1.19	888	0.246	453	511	598	738	888	1033	1160	1235	1283
**28**	118	1.12	1018	0.241	540	602	696	850	1017	1181	1327	1413	1468
**29**	102	1.06	1173	0.234	648	715	818	987	1173	1357	1522	1620	1684
**30**	157	1.01	1339	0.226	770	842	952	1136	1339	1543	1726	1836	1907
**31**	50	0.97	1515	0.216	904	980	1097	1294	1514	1735	1935	2055	2133
**32**	88	0.94	1709	0.206	1057	1138	1263	1473	1709	1947	2163	2293	2377
**33**	118	0.92	1927	0.195	1233	1319	1451	1675	1927	2181	2412	2551	2642
**34**	161	0.91	2160	0.183	1429	1519	1659	1894	2159	2428	2672	2819	2914
**35**	303	0.89	2399	0.172	1640	1734	1879	2123	2399	2678	2932	3086	3186
**36**	462	0.87	2633	0.161	1855	1950	2099	2350	2633	2920	3182	3340	3443
**37**	955	0.85	2851	0.150	2066	2162	2312	2565	2851	3142	3407	3567	3672
**38**	2183	0.80	3062	0.140	2280	2376	2524	2776	3062	3353	3619	3780	3886
**39**	4240	0.73	3230	0.131	2464	2557	2702	2949	3230	3518	3783	3944	4049
**40**	6223	0.65	3371	0.125	2610	2702	2845	3091	3371	3661	3928	4091	4198
**41**	5718	0.57	3514	0.121	2754	2845	2987	3232	3514	3806	4078	4244	4354
**42**	760	0.49	3590	0.117	2845	2934	3073	3313	3590	3879	4148	4314	4424
**43**	52	0.41	3630	0.113	2909	2994	3128	3360	3630	3912	4176	4339	4447

**Table 5:** Centiles for Girls, Multiparous

**Gestational Age**	**No**.	**L d.f. = 4**	**M d.f. = 14**	**S d.f. = 8**	**3^rd^**	**5^th^**	**10^th^**	**25^th^**	**50^th^**	**75^th^**	**90^th^**	**95^th^**	**97^th^**
**24**	65	1.14	630	0.247	326	366	426	524	630	734	826	881	916
**25**	59	1.11	720	0.245	377	422	489	599	719	837	942	1004	1044
**26**	101	1.09	832	0.244	442	492	568	694	832	968	1089	1161	1208
**27**	105	1.06	958	0.241	517	573	659	802	958	1114	1253	1335	1389
**28**	128	1.04	1073	0.235	595	656	748	902	1072	1242	1394	1484	1543
**29**	143	1.01	1222	0.225	706	770	870	1037	1222	1407	1574	1673	1738
**30**	160	0.96	1406	0.214	846	916	1023	1204	1406	1609	1793	1903	1975
**31**	86	0.92	1580	0.203	989	1061	1174	1365	1580	1798	1996	2115	2193
**32**	105	0.87	1759	0.193	1137	1213	1331	1531	1759	1990	2201	2329	2412
**33**	121	0.82	1956	0.184	1301	1381	1505	1716	1956	2202	2427	2564	2654
**34**	213	0.77	2188	0.177	1488	1572	1704	1930	2188	2453	2698	2847	2945
**35**	341	0.72	2442	0.173	1684	1775	1917	2161	2442	2732	3000	3164	3272
**36**	586	0.68	2687	0.169	1877	1973	2125	2386	2687	2999	3289	3466	3583
**37**	1051	0.65	2932	0.161	2092	2192	2349	2620	2932	3256	3558	3742	3864
**38**	2447	0.63	3176	0.146	2347	2446	2601	2868	3176	3495	3792	3974	4094
**39**	4459	0.62	3352	0.132	2556	2652	2801	3057	3352	3656	3939	4111	4225
**40**	6421	0.64	3498	0.126	2706	2802	2951	3206	3498	3799	4077	4247	4359
**41**	5906	0.69	3625	0.122	2824	2921	3072	3330	3625	3927	4205	4375	4486
**42**	867	0.73	3673	0.122	2855	2954	3109	3373	3673	3979	4260	4431	4543
**43**	55	0.78	3669	0.126	2821	2924	3085	3359	3668	3983	4271	4446	4560

**Figure 3:**
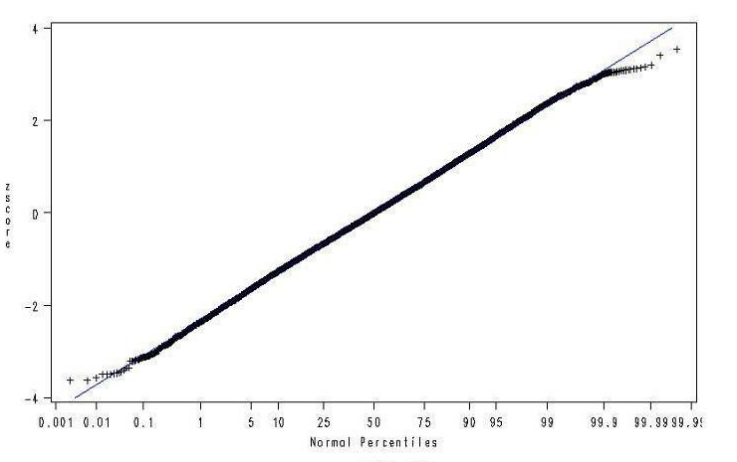
Q-Q plot of z-scores for girls, parity 1 or more.

The z-scores resulting from the LMS models fitted should be normally distributed within each grouping. This was verified by obtaining normal probability plots of the z-scores overall and for each gestational age. The plot for girls, parity 1 or more is given in Figure 3. Table 6 gives the observed percentage of z-scores by centiles groupings for the same group.

**Table 6:** Percentage of observations (observed and expected) within centile bands

**Centiles**	**Expected Percentage**	**Observed Percentage**
Less than 3^rd^	3	3.1
Between 3^rd ^and 5^th^	2	1.9
Between 5^th ^and 10^th^	5	4.8
Between 10^th ^and 25^th^	15	14.9
Between 25^th ^and 50^th^	25	25.5
Between 50^th ^and 75^th^	25	25.1
Between 75^th ^and 90^th^	15	14.5
Between 90^th ^and 95^th^	5	5.0
Between 95^th ^and 97^th^	2	2.0
Above 97^th^	3	3.2

**Figure 4:**
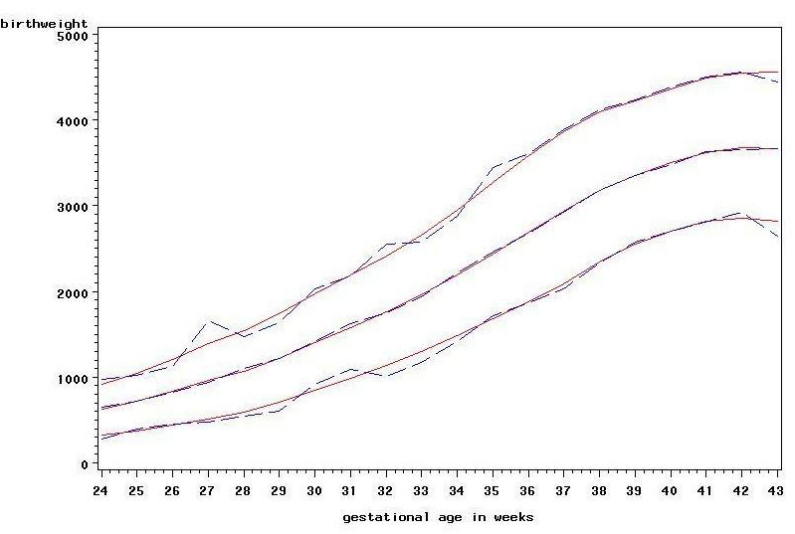
**New centiles vs observed centiles for girls, parity 1 or more.** 3^rd^, 50^th ^and 97^th ^centiles. Solid line: new centiles. Dashed line: observed centiles.

**Figure 5:**
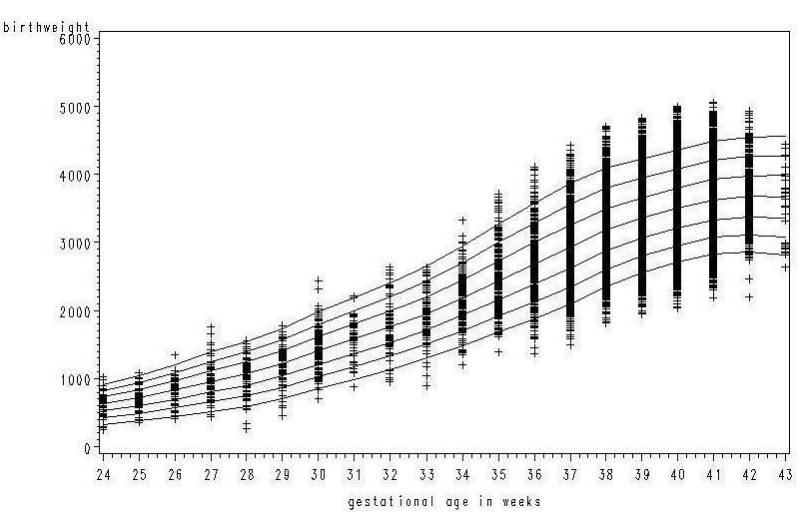
**New centiles with birthweights for girls, parity 1 or more**. 3^rd^, 10^th^, 25^th^, 50^th^, 75^th^, 90^th^, 97^th ^centiles.

**Figure 6:**
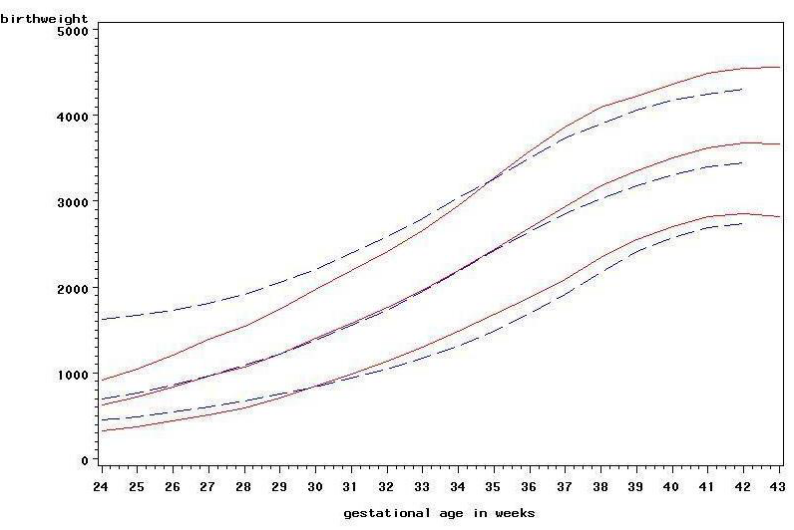
**New centiles vs ISD centiles for girls, parity 1 or more.** 3^rd^, 50^th ^and 97^th ^centiles. Solid line: new centiles. Dashed line: ISD centiles.

In order to assess the goodness of fit of the models, the new centiles were plotted against the observed centiles for each group. Figure 4 shows this plot for the 3^rd^, 50^th ^and 97^th ^centiles for girls, parity 1 or more. Figure 5 shows the 3^rd^, 10^th^, 25^th^, 50^th^, 75^th^, 90^th ^and 97^th ^centiles superimposed on the actual birthweights for the same groups. For comparison Figure 6 shows the new centiles compared to the currently used ISD centiles again for the 3^rd^, 50^th ^and 97^th ^centiles

## Discussion

Centile charts of birthweight for gestational age are a valuable tool in many epidemiological studies as well as providing important information to clinicians as to which babies may be at higher risk of neonatal or postnatal morbidity [14]. It is therefore essential that the charts used are representative of the population to which they are applied. A number of standards are available based on births occurring in various European countries; mostly using data from the 1980s and the 1990s [15].

There are clear differences between the centiles calculated here from recent data and those in current use in Scotland which are based on data from 1975–1989. For term babies the median birthweight in all sex and parity groupings is shown to be higher than it was previously. This increase in birthweight is also reflected in the other centiles. For babies born at very low gestational ages the median birthweight is now less than it was, possibly reflecting the increased survival rate in pre-term births [16]. The centiles for lower gestational ages are also much closer together than in the existing charts. One possible explanation for such a marked difference at lower gestations in particular may be poor estimation of gestational age, particularly in the 1970s, as was found in data for England and Wales analysed by Milner and Richards in 1974 [17].

In recent years a number of centiles charts [18] have been constructed using the method developed by Gardosi [19]. This method aims to give a fetal weight standard and requires only data for term births from the population of interest. Whilst it is desirable in principle to look at fetal weights the assumptions which are being made with this method cannot be substantiated with reference to our data which consists only of actual birthweights. It is therefore not possible to assess the goodness of fit of the centiles calculated in this way.

As well as modelling the median birthweight the LMS method also models the coefficient of variation S and the power L which is used to transform the birthweights to achieve normality. Within each of the sex and parity groupings it is seen that the coefficient of variation decreases with increased gestational age showing that the birthweights are more variable at lower gestational ages. This contrasts with the assumption used in Gardosi's methodology for fetal weights that the coefficient of variation is constant.

It is important in constructing charts of this type to test the adequacy of the model fitted both with reference to the raw data used to construct the charts and to the assumptions on which the model relies [20,21].

Comparing the new centiles to the empirical centiles suggests that the LMS method is a reasonable fit to the data. It can be shown that, in general, the standard errors of empirical centiles are larger than those for the centiles calculated using the normal distribution. The latter method is therefore more efficient. This is only true if the assumption of normality is reasonable which is not the case for birthweight and therefore some transformation of the data is required. A value of 1 for L indicates no transformation required with a value less than 1 adjusting for positive skewness and a value greater than 1 for negative skewness. For each sex and parity grouping the values of L suggest that the birthweights are negatively skewed for low gestational ages and positively skewed for higher gestational ages. The values of L suggest the extent of skewness at each gestational age is not high.

Normal probability plots of the z-scores for each grouping and for each gestational age within each grouping show that the LMS method has largely succeeded in achieving normality. There is some suggestion in the plot of heavier tails however the percentages in the tails are close to what is expected.

An important question in constructing centile charts of any data is which cases to include in the calculations and which to omit. Many previous studies into centile charts have used live births only because of the difficulty of accurately assessing the gestational age of stillbirths. The argument in the past has been that a baby which is stillborn may have died some time before delivery and therefore the weight may not be a true reflection of the gestational age at which delivery occurs. This is not often the case now. Fetal death is almost always recognized very quickly, and most women prefer to be delivered as soon as possible once it is realized that this has happened. This was argued by Tin[16] looking at the problems of estimating centiles for babies born before 32 weeks gestational age, In this paper it was suggested that not all stillbirths should be excluded, arguing that by doing so centiles at gestational ages less than 28 weeks have been largely overestimated.

For babies born within ten weeks of term the difference in centiles including and excluding stillbirths are negligible because the numbers of stillbirths are relatively small. Omitting stillbirths at low gestations of 24–27 weeks gestation causes bias in the centiles possibly because very small babies at any specified gestation are much more likely to be treated as "effectively" stillborn than larger babies of the same gestation when pregnancy ends as soon as this.

Information on ethnicity is poorly recorded on the SMR02 forms therefore no attempt was made to produce separate centiles for different ethnic groups. From the 2001 census it is known that the minority ethnic population was just over 100,000 in that year which is 2% of the total population of Scotland. The percentage is similar for women of child bearing age. Ethnicity is not therefore a major consideration for the Scottish data.

Other studies have followed the convention of excluding babies with major congenital malformations [22] and this has been used in this study. However with such a large data set the exclusion has made little difference to the centiles. Other studies [1,23] have also identified outliers at each gestational age using the criterion outlined by Tukey. From visual inspection of the charts with and without the outliers, it is clear that the points identified in this study are most likely to be due to transcription errors. The excluded points do not therefore raise any concerns about the accuracy with which gestational age is measured.

Other factors are known to have a significant effect on birthweight and a number of customised charts have been developed in recent years. It can be desirable to take into account physiological factors such as the height of the mother which contribute to the natural variation in birthweights but not potential risk factors such as whether or not the mother smokes. The distinction between the two types of factor may not always be clear cut however. For example height and weight of the mother may in part be determined by risk factors such as social deprivation or nutrition. There is therefore an important role in epidemiological studies into adverse perinatal outcomes for charts such as the ones described here which will allow both the effect of infant's size and the size of the mother to be separated.

## Conclusion

The differences shown between the new centiles and the current published centiles confirm the need to have centiles appropriate for the population for which the charts are to be used. Use of inappropriate centiles may result either in small-for-dates babies not being identified or too many babies being flagged as small-for-dates. After consistent increases in mean birthweight from 1980 until the mid 1990s, mean birthweight has stabilised over recent years making the new charts appropriate for current use. It is however important that the distribution of birthweight continues to be monitored on a regular basis.

## Competing interests

The author(s) declare that they have no competing interests.

## Authors' contributions

SRB and CW carried out the statistical analysis. All authors were co-investigators on the CSO grant and contributed to the initiation of the project and subsequent discussion. All authors have read and approved the final manuscript.

## Pre-publication history

The pre-publication history for this paper can be accessed here:


